# Tools and techniques to identify, study, and control *Candida auris*

**DOI:** 10.1371/journal.ppat.1011698

**Published:** 2023-10-19

**Authors:** James Carty, Anuradha Chowdhary, Douglas Bernstein, Shankar Thangamani

**Affiliations:** 1 Department of Biology, Ball State University, Muncie, Indiana, United States of America; 2 Medical Mycology Unit, Department of Microbiology, Vallabhbhai Patel Chest Institute, University of Delhi, Delhi, India; 3 National Reference Laboratory for Antimicrobial Resistance in Fungal Pathogens, Vallabhbhai Patel Chest Institute, University of Delhi, Delhi, India; 4 Department of Comparative Pathobiology, College of Veterinary Medicine, Purdue University, West Lafayette, Indiana, United States of America; 5 Purdue Institute for Immunology, Inflammation and Infectious Diseases (PI4D), Indiana, United States of America; University of Maryland, Baltimore, UNITED STATES

## Abstract

*Candida auris*, is an emerging fungal pathogen that can cause life-threatening infections in humans. Unlike many other *Candida* species that colonize the intestine, *C*. *auris* most efficiently colonizes the skin. Such colonization contaminates the patient’s environment and can result in rapid nosocomial transmission. In addition, this transmission can lead to outbreaks of systemic infections that have mortality rates between 40% and 60%. *C*. *auris* isolates resistant to all known classes of antifungals have been identified and as such, understanding the underlying biochemical mechanisms of how skin colonization initiates and progresses is critical to developing better therapeutic options. With this review, we briefly summarize what is known about horizontal transmission and current tools used to identify, understand, and control *C*. *auris* infections.

## Introduction

*Candida auris*, is an emerging multidrug-resistant human fungal pathogen that predominately colonizes the skin. *C*. *auris* has been classified as an urgent threat by the US Centers for Disease Control and Prevention (CDC) Antibiotic Threats Report (2019) and ranked in the critical priority group by the World Health Organization (WHO) in a recently released list of fungal priority pathogens [1,2]. *C*. *auris* is found in acute care settings and long-term care facilities and where it can spread leading to systemic infections [3–6]. In some regions such as in India and parts of South Africa, *C*. *auris* has now surpassed *Candida albicans* as the most common *Candida* bloodstream species with the mortality rate ranging from 40% to 60% among infected patients [7,8]. Risk factors for *C*. *auris* colonization and infection include extensive exposure to healthcare; advanced age; chronic medical conditions including diabetes mellitus, chronic renal disease, and immune compromise; and exposure to broad-spectrum antibiotics [4,9]. Several isolates of *C*. *auris* have been identified that exhibit resistance to the azoles, polyenes, and echinocandins, the 3 major classes of FDA-approved antifungal drugs. The ability of *C*. *auris* to spread quickly in medical setting in coordination with this potential drug resistance poses significant challenges when attempting to treat infections [8,10,11]. *C*. *auris* isolates were mainly classified under 4 clades based on whole genome sequencing data of clinical strains [8]. Differences in distribution, virulence, and phenotypic properties between different clades were recently studied (Table 1).

**Table 1:** Differences between 4 clades of *C*. *auris*.

	Clade I (South Asian)	Clade II (East Asian)	Clade III (South African)	Clade IV (South American)	Refs
**Distribution and hospital outbreaks**	Canada, France, Germany, India, Kenya, Pakistan, Saudi Arabia, United Kingdom, United Arab Emirates, and United States	Canada, Japan, South Korea, and United States	Australia, Canada, Kenya, South Africa, Spain, and United States	Colombia, Israel, Panama, United States, and Venezuela	[12]
**Percent mortality in neutropenic mice**	80	44	45	96	[13]
**Percent resistance to at least 2 drug families**	53	0	4	12	[12]
**Morphology**	Yeast Filamentous Elongated Weakly aggregated	Yeast Filamentous Weakly aggregated	Yeast Filamentous Elongated Aggregated	Yeast Filamentous Elongated Aggregated	[14,15]
**Infection site**	Invasive skin Respiratory and urinary tract	Ear canal Rarely invasive	Invasive skin Respiratory and urinary tract	Invasive skin Respiratory and urinary tract	[16,17]
**Murine skin colonization**	++	+	++++	+++	[18]
**Murine skin residency**	++	+	++++	+++	[18]
**Carbon utilization**	α-D-Glucose D-Mannose D-Fructose Dihydroxyacetone 2-Deoxy-D-Ribose Sucrose, D-Trehalose D-Glucosamine 5-Keto-D-Gluconic Acid Oxalomalic Acid GlcNAc Melibionic acid	α-D-Glucose D-Mannose D-Fructose Dihydroxyacetone 2-Deoxy-D-Ribose Sucrose D-Trehalose D-Glucosamine 5-Keto-D-Gluconic Acid Oxalomalic Acid	α-D-Glucose D-Mannose D-Fructose Dihydroxyacetone 2-Deoxy-D-Ribose Sucrose D-Trehalose D-Glucosamine 5-Keto-D-Gluconic Acid, Oxalomalic Acid, GlcNAc	α-D-Glucose D-Mannose D-Fructose Dihydroxyacetone 2-Deoxy-D-Ribose Sucrose D-Trehalose D-Glucosamine 5-Keto-D-Gluconic Acid Oxalomalic Acid, GlcNAc Melibionic acid d-tagatose	[19]
**Nitrogen utilization**	L-Glutamine Dipeptides–His Dipeptides–Ala Dipeptides–Gln Ammonia l-valine l-isoleucine l-lysine l-leucine l-phenylalanine l-tryptophan	L-Glutamine Dipeptides–Ala Dipeptides–His Dipeptides–Gln Ammonia	L-Glutamine Dipeptides–His Dipeptides–Ala Dipeptides–Gln Ammonia	L-Glutamine Dipeptides–His Dipeptides–Ala Dipeptides–Gln Ammonia	[19]
**Osmotic stress tolerance**	NaCl ≤ 8% KCL ≤ 6% HCOONa ≤ 2% NaC3H5O3 ≤ 4%	NaCl ≤ 3% KCL ≤ 4% NaC3H5O3 ≤ 4%	NaCl ≤ 8% KCl ≤ 6% NaC3H5O3 ≤ 4%	NaCl ≤ 3% KCL ≤ 4% NaC3H5O3 ≤ 3%	[19]
**Mating type**	MTLa–a1, a2	MTLα – α1	MTLα – α1	MTLa–a1, a2	[20]

### How does *C*. *auris* spread in healthcare settings?

Unlike many other *Candida* species, *C*. *auris* efficiently colonizes the skin leading to contamination of the patient’s environment [21]. This phenotype likely plays a role in *C*. *auris’* ability to cause healthcare-associated outbreaks. *C*. *auris* can colonize nares, palms, fingertips, axilla, inguinal crease, and toe webs [3,22], and colonization can lead to contamination of surfaces including floors, bed rails, bed sheet, bed hand controllers, bed side trolley, mobile phones, chairs, bed trays, air conditioning wings, and sinks. *C*. *auris* can persist for up to 2 weeks on dry surfaces and it has been detected on surfaces with little to no patient contact and infrequent healthcare worker contact such as closet cabinets, door handles, and alcohol gel dispensers [21]. Medical equipment that comes in contact with patients has also been linked to transmission as *C*. *auris* has been detected on temperature probes, blood pressure cuffs, glucometers, intravenous poles, oxygen masks, housekeeping carts, dialysis equipment, ultrasound machines, computer monitors, and keypads [22,23]. While these findings suggest the contaminated inanimate environment plays a role in cross-transmission of *C*. *auris* in healthcare settings, the relative contribution of direct transmission via fomites, patient-to-patient, or environment-to-patient transmission via healthcare provider have not yet been defined. Further studies are essential to understand what contributes to *C*. *auris’s* persistence in the environment and how this leads to infection.

### How do we identify *C*. *auris* isolates on the patient and in the environment?

*C*. *auris* infections can present similarly to other fungal infections, and drug-resistant fungal strains can phenotypically behave like antibiotic susceptible isolates when not challenged with drug. As such, it is critical the medical community have the ability to detect *C*. *auris* on the skin and quickly determine a particular culture’s antifungal susceptibility. A variety of PCR-based assays have been established to identify *C*. *auris* in the healthcare environment and in waste water [24,25] and real-time PCR and loop-mediated isothermal amplification-based assays have been developed to screen for *C*. *auris* on patient’s skin [26,27]. In addition, standard PCR-based assays that do not require advanced infrastructure can probe for *C*. *auris*-specific GPI protein encoding genes [28]. As PCRs take a couple of hours to run and process, these tests provide relatively quick surveillance of an environment or patient. In addition, T2 Magnetic Resonance system-based assays originally designed as rapid diagnostic platform to detect pathogens in blood samples have also been developed to detect *C*. *auris* 5 CFU/ml from the skin [29].

The combination of efficient environmental and patient surveillance testing is critical to the control and effective treatment of *C*. *auris* in medical facilities. One, current limitation of these assays is that drug resistance, for the most part, cannot be determined using these techniques. *C*. *auris* has been shown to use a variety of mechanisms including changes in ploidy and transcription to quickly alter drug susceptibility [30,31]. As such, traditional growth assays must be used to determine antifungal susceptibility. As we learn more about *C*. *auris* drug resistance mechanisms, developing quick molecular tests such as assays to detect *FKS1* mutations to determine susceptibility to echinocandins can help in the future [32].

### How can we genetically manipulate *C*. *auris*?

*C*. *auris*, along with human pathogens *C*. *albicans*, *C*. *tropicalis*, and *C*. *parapsilosis* are members of the CTG fungal clade where the CTG codon is primarily translated as serine as opposed to leucine [33]. *C*. *auris* displays high salt tolerance that potentially plays a role in its ability to colonize the high salt environment of the skin. In addition, isolates of *C*. *auris* have been shown to be naturally resistant to many antifungal compounds distinguishing it from other CTG species [34]. Analysis of the *C*. *auris* genome sequence suggests that copy number variation and mutation of transporters, lipases, and drug targets could play roles in these phenotypes [20]. The efficient and accurate manipulation of the *C*. *auris* genome is critical to understanding the underlying mechanisms that support such phenotypes. Since the *C*. *auris* was genome sequenced, a number of methodologies have been developed to determine the origins of these phenotypes [35]. Forward genetic screens using *Agrobacterium*-mediated transformation (AtMT) have successfully been employed on *C*. *auris* [36]. In AtMT, a nourseothricin (NAT^r^*)* selectable marker is incorporated into the *Agrobacterium tumefaciens* Ti plasmid. This is then transformed into *A*. *tumefaciens* strain EHA105 that contains the virulence genes required for recruitment of the T-DNA. Co-culturing the modified *A*. *tumefaciens* and *C*. *auris* results in gene disruption by the plasmid while conferring nourseothricin resistance. *C*. *auris* transformants are selected based on resultant phenotypes, and whole genome sequencing is completed to identify disrupted genes. This approach has been used to screen for genes important for cellular morphology. Mutants responsible for changes in morphology were recovered in all 4 clades at rates comparable to experiments performed in other yeast species [37]. Once genes of interest are identified, reverse genetics approaches can be applied to confirm the role of identified genes in the observed phenotype.

Investigators have a number of choices when applying reverse genetics to interrogate *C*. *auris*. As has been done with a variety of other fungi, direct homologous recombination methods have been employed with success to disrupt genes of interest [38]. In addition, CRISPR-Cas9 systems have also been applied to *C*. *auris*. These systems must be incorporated into the *C*. *auris* genome, as to date *C*. *auris* has not been shown to be able to maintain plasmids. The permanent incorporation of CRISPR components and selective markers to the genome presents a challenge as these components can limit the ability to further genetically manipulate a strain. Investigators have sought to mitigate this challenge in a variety of ways. Direct transformation of CRISPR Cas9 RNA protein complexes into *C*. *auris* using electroporation can be used to edit the *C*. *auris* genome [39]. Furthermore, recyclable CRISPR-mediated systems that leverage lithium acetate transformation protocols have been optimized for *C*. *auris*. Cas9/sgRNA cassettes marked with either Nat^r^ or hygromycin^r^ also containing regions of homology to the *LEU2* locus facilitate insertion of CRISPR components to the genome while simultaneously disrupting *LEU2* [40]. Cells that have incorporated the CRISPR machinery will grow on drug but require leucine. Once CRISPR-mediated genome editing has been completed, transformants are plated on synthetic complete media lacking leucine to select for the reconstitution of *LEU2* by homologous recombination and resultant recycling of Cas9-sgRNA insertions from the genome, thus enabling investigators to serially edit the *C*. *auris* genome.

### What model organisms can we use to study *C*. *auris* pathogenesis?

Animal models that accurately recapitulate *C*. *auris* infection are critical to understanding pathogenesis. Although there are differences in skin biology between humans and mice [41], mouse models offer numerous advantages when studying virulence. Genetically modified animals and well-characterized immunological tools are available and are widely used to understand skin infection pathogenesis in vivo [18]. For example, a recent study by Huang and colleagues utilized knock out mouse models to dissect the contribution of IL-17-mediated innate and adaptive immune response in *C*. *auris* skin colonization [18]. Furthermore, recent studies compared the virulence potential of *C*. *auris* and other *Candida* species including *C*. *albicans* using immunocompetent and immunocompromised mice models of systemic infection [13,42,43]. *C*. *auris* elicits less immunoinflammatory response than *C*. *albicans*, and this differential response correlates with distinctions in cell wall structure [43]. *C*. *albicans* is a commensal yeast and it is likely its surface determinants have coevolved with the human immune system. *C*. *auris* is not an obligate commensal, but can colonize different host niches including skin, gut, and internal organs [18,43,44]. Comparing differences between the immune response to these pathogens have the potential to provide insights into how virulence mechanisms between these pathogens differ.

Aside from mouse, other model systems such as Zebrafish [45], *Galleria mellonella* [46], *Caenorhabditis elegans* [47], and *Drosophila melanogaster* [48] are utilized to study *C*. *auris* colonization and pathogenesis and can provide distinct advantages to mammalian models. Furthermore, ex vivo models such as human and pig skin can be used to conduct *C*. *auris* colonization and biofilm studies [41,49–51]. Collectively, depending on the type of experiment, invertebrate whole animal, mouse, and ex vivo skin models can be utilized to investigate fungal virulence and host–pathogen interactions.

### What are the current decolonization and disinfection strategies used to control *C*. *auris*?

Decolonization in patients and environmental decontamination of *C*. *auris* is critical to prevent and control the spread of *C*. *auris* infections. Currently, there is no effective skin decolonization strategy for *C*. *auris*. Chlorhexidine gluconate (CHG) skin wash is used in some facilities to reduce the skin burden of multidrug-resistant microorganisms including *C*. *auris*. However, only high concentrations of CHG (≥625 μg/ml), which are rarely achieved on the skin, were shown to be effective for reducing the burden of *C*. *auris* on the skin in 1 study [3]. Persistent colonization has been reported in spite of using this antiseptic [3,52,53]. Further, using effective disinfectants is critical for the prevention and control of *C*. *auris* in the environment. The most current CDC recommendations for infection control can be found on the CDC website and include a list of recommended environmental disinfectants and other recommended practices [54]. These recommendations for disinfectants were based on recent studies showing efficacy of hydrogen peroxide or alcohol-based active compounds against *C*. *auris* and ineffectiveness of products based on quaternary ammonium chemistry (QAC) [55,56]. In addition, recently *C*. *auris* was also detected in the wastewater samples in Nevada, United States of America [25]. Further studies are needed to develop novel strategies to evaluate the efficacy of currently available disinfectants and control measures in real-world settings.
